# 4-(2*H*-Tetra­zol-5-yl)pyridinium perchlorate

**DOI:** 10.1107/S1600536809018200

**Published:** 2009-05-20

**Authors:** Jing Dai

**Affiliations:** a Ordered Matter Science Research Center, College of Chemistry and Chemical Engineering, Southeast University, Nanjing 210096, People’s Republic of China

## Abstract

In the cation of the title compound, C_6_H_6_N_5_^+^·ClO_4_^−^, the pyridinium and tetra­zole rings form a dihedral angle of 23.6 (1)°. In the crystal structure, weak inter­molecular N—H⋯O and N—H⋯N hydrogen bonds link cations and anions into chains extending along the *b* axis.

## Related literature

For applications of tetra­zole derivatives in coordination chemistry, see: Xiong *et al.* (2002 ▶); Wang *et al.* (2005 ▶). For related structures, see: Dai & Fu (2008 ▶); Wen (2008 ▶).
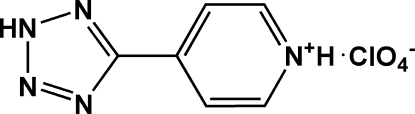


## Experimental

### 

#### Crystal data

C_6_H_6_N_5_^+^·ClO_4_^−^
*M* *_r_* = 247.61
Monoclinic, 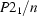

*a* = 5.2033 (10) Å
*b* = 14.764 (3) Å
*c* = 12.244 (2) Å
β = 101.78 (3)°
*V* = 920.8 (3) Å^3^
*Z* = 4
Mo *K*α radiation
μ = 0.43 mm^−1^
*T* = 298 K
0.30 × 0.25 × 0.20 mm

#### Data collection

Rigaku Mercury2 diffractometer
Absorption correction: multi-scan (*CrystalClear*; Rigaku, 2005 ▶) *T*_min_ = 0.872, *T*_max_ = 1.000 (expected range = 0.801–0.919)
9546 measured reflections
2108 independent reflections
1849 reflections with *I* > 2σ(*I*)
*R*_int_ = 0.036

#### Refinement

*R*[*F* ^2^ > 2σ(*F* ^2^)] = 0.035
*wR*(*F* ^2^) = 0.093
*S* = 1.09
2108 reflections
146 parameters
H-atom parameters constrained
Δρ_max_ = 0.29 e Å^−3^
Δρ_min_ = −0.37 e Å^−3^

### 

Data collection: *CrystalClear* (Rigaku, 2005 ▶); cell refinement: *CrystalClear*; data reduction: *CrystalClear*; program(s) used to solve structure: *SHELXS97* (Sheldrick, 2008 ▶); program(s) used to refine structure: *SHELXL97* (Sheldrick, 2008 ▶); molecular graphics: *SHELXTL* (Sheldrick, 2008 ▶); software used to prepare material for publication: *SHELXTL*.

## Supplementary Material

Crystal structure: contains datablocks I, global. DOI: 10.1107/S1600536809018200/cv2545sup1.cif

Structure factors: contains datablocks I. DOI: 10.1107/S1600536809018200/cv2545Isup2.hkl

Additional supplementary materials:  crystallographic information; 3D view; checkCIF report

## Figures and Tables

**Table 1:** Hydrogen-bond geometry (Å, °)

*D*—H⋯*A*	*D*—H	H⋯*A*	*D*⋯*A*	*D*—H⋯*A*
N5—H5*A*⋯O3^i^	0.86	2.28	2.964 (2)	136
N5—H5*A*⋯N2^ii^	0.86	2.38	3.059 (2)	136
N3—H3*A*⋯O4^iii^	0.86	2.21	2.884 (2)	135

Symmetry codes: (i) 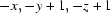; (ii) 
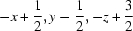
; (iii) 
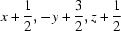
.

**Fig. 1.**
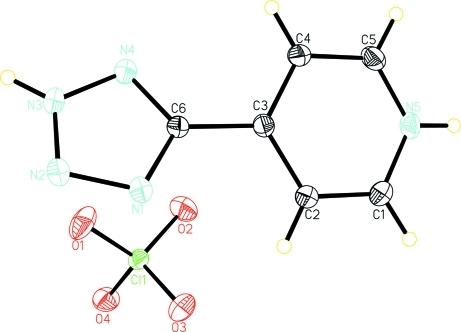
A view of the title compound with the atomic numbering scheme. Displacement ellipsoids were drawn at the 30% probability level.

## supplementary crystallographic information

### Comment

In the past few years, more and more people have focused their attention on the chemistry of tetrazole derivatives because of their multiple coordination modes as ligands to metal ions and for the construction of novel metal-organic frameworks (Wang *et al.* 2005; Xiong *et al.* 2002; Wen, 2008). We report here the crystal structure of the title compound, 4-(2*H*-tetrazol-5-yl)pyridinium perchlorate).

In the title compound (Fig.1), the pyridine N atom is protonated. The pyridine ring makes a dihedral angle of 23.62 (1)° with the tetrazole ring. The geometric parameters of the tetrazole rings are comparable to those in related molecules (Wang *et al.* 2005; Dai & Fu, 2008).

The crystal packing is stabilized by N—H···O and N—H···N hydrogen bonds (Table 1) with the formation of zig-zag chains parallel to *b* axis.

### Experimental

Isonicotinonitrile (30 mmol), NaN _3_ (45 mmol), NH_4_Cl (33 mmol) and DMF (50 ml) were added in a flask under nitrogen atmosphere and the mixture stirred at 110°C for 20 h. The resulting solution was then poured into ice-water (100 ml), and a white solid was obtained after adding HCl (6 *M*) till pH=6. The precipitate was filtered and washed with distilled water. Colourless block-shaped crystals suitable for X-ray analysis were obtained from the crude product by slow evaporation of an ethanol/HClO_4_ (50:1 *v*/*v*) solution.

### Refinement

All H atoms attached to C and N atoms were fixed geometrically and treated as riding with C–H = 0.93 Å (aromatic) and N–H = 0.86 Å with *U*_iso_(H) =1.2Ueq(C or N).

### Crystal data

**Table:**

C_6_H_6_N_5_^+^·ClO_4_^−^	*F*(000) = 504
*M**_r_* = 247.61	*D*_x_ = 1.786 Mg m^−^^3^
Monoclinic, *P*2_1_/*n*	Mo *K*α radiation, λ = 0.71073 Å
Hall symbol: -P 2yn	Cell parameters from 2108 reflections
*a* = 5.2033 (10) Å	θ = 3.2–27.5°
*b* = 14.764 (3) Å	µ = 0.43 mm^−^^1^
*c* = 12.244 (2) Å	*T* = 298 K
β = 101.78 (3)°	Block, colourless
*V* = 920.8 (3) Å^3^	0.30 × 0.25 × 0.20 mm
*Z* = 4	

### Data collection

**Table:**

Rigaku Mercury2 diffractometer	2108 independent reflections
Radiation source: fine-focus sealed tube	1849 reflections with *I* > 2σ(*I*)
graphite	*R*_int_ = 0.036
Detector resolution: 13.6612 pixels mm^-1^	θ_max_ = 27.5°, θ_min_ = 3.2°
CCD profile fitting scans	*h* = −6→6
Absorption correction: multi-scan (*CrystalClear*; Rigaku, 2005)	*k* = −19→19
*T*_min_ = 0.872, *T*_max_ = 1.000	*l* = −15→15
9546 measured reflections	

### Refinement

**Table:**

Refinement on *F*^2^	Secondary atom site location: difference Fourier map
Least-squares matrix: full	Hydrogen site location: inferred from neighbouring sites
*R*[*F*^2^ > 2σ(*F*^2^)] = 0.035	H-atom parameters constrained
*wR*(*F*^2^) = 0.093	*w* = 1/[σ^2^(*F*_o_^2^) + (0.0391*P*)^2^ + 0.4636*P*] where *P* = (*F*_o_^2^ + 2*F*_c_^2^)/3
*S* = 1.09	(Δ/σ)_max_ < 0.001
2108 reflections	Δρ_max_ = 0.29 e Å^−^^3^
146 parameters	Δρ_min_ = −0.37 e Å^−^^3^
0 restraints	Extinction correction: *SHELXL97* (Sheldrick, 2008), Fc^*^=kFc[1+0.001xFc^2^λ^3^/sin(2θ)]^-1/4^
Primary atom site location: structure-invariant direct methods	Extinction coefficient: 0.032 (3)

### Special details

**Table:**

Geometry. All e.s.d.'s (except the e.s.d. in the dihedral angle between two l.s. planes) are estimated using the full covariance matrix. The cell e.s.d.'s are taken into account individually in the estimation of e.s.d.'s in distances, angles and torsion angles; correlations between e.s.d.'s in cell parameters are only used when they are defined by crystal symmetry. An approximate (isotropic) treatment of cell e.s.d.'s is used for estimating e.s.d.'s involving l.s. planes.
Refinement. Refinement of *F*^2^ against ALL reflections. The weighted *R*-factor *wR* and goodness of fit *S* are based on *F*^2^, conventional *R*-factors *R* are based on *F*, with *F* set to zero for negative *F*^2^. The threshold expression of *F*^2^ > σ(*F*^2^) is used only for calculating *R*-factors(gt) *etc*. and is not relevant to the choice of reflections for refinement. *R*-factors based on *F*^2^ are statistically about twice as large as those based on *F*, and *R*- factors based on ALL data will be even larger.

### Fractional atomic coordinates and isotropic or equivalent isotropic displacement parameters (Å^2^)

**Table:**

	*x*	*y*	*z*	*U*_iso_*/*U*_eq_	
Cl1	0.54774 (8)	0.68556 (3)	0.60933 (3)	0.02861 (15)	
O4	0.6154 (3)	0.76134 (10)	0.54624 (12)	0.0425 (4)	
O3	0.2704 (3)	0.68626 (10)	0.60633 (13)	0.0433 (4)	
O2	0.6150 (3)	0.60331 (10)	0.55912 (15)	0.0524 (4)	
N1	0.3679 (3)	0.59831 (10)	0.88383 (13)	0.0307 (3)	
N5	0.0708 (3)	0.33991 (11)	0.61681 (12)	0.0317 (3)	
H5A	−0.0101	0.3029	0.5673	0.038*	
N4	0.7318 (3)	0.51759 (10)	0.89177 (13)	0.0325 (4)	
C3	0.3301 (3)	0.45759 (11)	0.77061 (13)	0.0247 (3)	
C6	0.4761 (3)	0.52315 (11)	0.85006 (13)	0.0246 (3)	
N2	0.5614 (3)	0.64262 (10)	0.94760 (13)	0.0327 (4)	
C5	0.2877 (4)	0.31159 (12)	0.68590 (16)	0.0337 (4)	
H5	0.3471	0.2526	0.6810	0.040*	
N3	0.7722 (3)	0.59329 (10)	0.95023 (13)	0.0325 (4)	
H3A	0.9246	0.6090	0.9871	0.039*	
O1	0.6915 (3)	0.69193 (12)	0.72169 (12)	0.0516 (4)	
C2	0.0996 (3)	0.48426 (12)	0.69840 (15)	0.0298 (4)	
H2	0.0327	0.5423	0.7026	0.036*	
C4	0.4234 (4)	0.37000 (12)	0.76447 (15)	0.0308 (4)	
H4	0.5756	0.3511	0.8129	0.037*	
C1	−0.0268 (4)	0.42336 (13)	0.62097 (15)	0.0334 (4)	
H1	−0.1799	0.4401	0.5716	0.040*	

### Atomic displacement parameters (Å^2^)

**Table:**

	*U*^11^	*U*^22^	*U*^33^	*U*^12^	*U*^13^	*U*^23^
Cl1	0.0264 (2)	0.0283 (2)	0.0283 (2)	−0.00122 (15)	−0.00104 (16)	0.00377 (16)
O4	0.0439 (8)	0.0389 (8)	0.0435 (8)	−0.0015 (6)	0.0060 (6)	0.0151 (6)
O3	0.0268 (7)	0.0536 (9)	0.0482 (8)	−0.0018 (6)	0.0044 (6)	−0.0002 (7)
O2	0.0510 (9)	0.0366 (8)	0.0687 (11)	0.0080 (7)	0.0098 (8)	−0.0074 (7)
N1	0.0302 (8)	0.0249 (8)	0.0349 (8)	−0.0011 (6)	0.0013 (6)	−0.0052 (6)
N5	0.0349 (8)	0.0290 (8)	0.0287 (8)	−0.0066 (6)	0.0006 (6)	−0.0077 (6)
N4	0.0284 (8)	0.0285 (8)	0.0371 (8)	0.0002 (6)	−0.0016 (6)	−0.0049 (6)
C3	0.0260 (8)	0.0227 (8)	0.0247 (8)	−0.0023 (6)	0.0036 (6)	−0.0002 (6)
C6	0.0262 (8)	0.0215 (8)	0.0244 (8)	0.0000 (6)	0.0012 (6)	0.0007 (6)
N2	0.0346 (8)	0.0266 (8)	0.0344 (8)	−0.0041 (6)	0.0014 (6)	−0.0049 (6)
C5	0.0423 (11)	0.0224 (9)	0.0348 (10)	0.0007 (7)	0.0037 (8)	−0.0026 (7)
N3	0.0289 (8)	0.0293 (8)	0.0348 (8)	−0.0038 (6)	−0.0038 (6)	−0.0047 (6)
O1	0.0471 (9)	0.0699 (11)	0.0306 (8)	−0.0147 (7)	−0.0092 (6)	0.0103 (7)
C2	0.0291 (9)	0.0249 (9)	0.0328 (9)	0.0015 (7)	0.0003 (7)	−0.0014 (7)
C4	0.0331 (9)	0.0247 (9)	0.0304 (9)	0.0024 (7)	−0.0030 (7)	−0.0009 (7)
C1	0.0294 (9)	0.0345 (10)	0.0325 (9)	−0.0008 (7)	−0.0027 (7)	−0.0011 (8)

### Geometric parameters (Å, °)

**Table:**

Cl1—O1	1.4285 (15)	C3—C4	1.389 (2)
Cl1—O2	1.4363 (15)	C3—C2	1.394 (2)
Cl1—O3	1.4363 (15)	C3—C6	1.469 (2)
Cl1—O4	1.4433 (14)	N2—N3	1.312 (2)
N1—N2	1.315 (2)	C5—C4	1.375 (2)
N1—C6	1.347 (2)	C5—H5	0.9300
N5—C5	1.332 (2)	N3—H3A	0.8600
N5—C1	1.338 (2)	C2—C1	1.373 (2)
N5—H5A	0.8600	C2—H2	0.9300
N4—N3	1.321 (2)	C4—H4	0.9300
N4—C6	1.327 (2)	C1—H1	0.9300
			
O1—Cl1—O2	110.02 (11)	N3—N2—N1	105.86 (15)
O1—Cl1—O3	110.48 (10)	N5—C5—C4	119.66 (17)
O2—Cl1—O3	109.06 (9)	N5—C5—H5	120.2
O1—Cl1—O4	109.12 (9)	C4—C5—H5	120.2
O2—Cl1—O4	108.60 (10)	N2—N3—N4	114.64 (14)
O3—Cl1—O4	109.53 (9)	N2—N3—H3A	122.7
N2—N1—C6	105.96 (15)	N4—N3—H3A	122.7
C5—N5—C1	122.96 (15)	C1—C2—C3	118.82 (16)
C5—N5—H5A	118.5	C1—C2—H2	120.6
C1—N5—H5A	118.5	C3—C2—H2	120.6
N3—N4—C6	101.09 (14)	C5—C4—C3	119.14 (16)
C4—C3—C2	119.59 (15)	C5—C4—H4	120.4
C4—C3—C6	120.69 (15)	C3—C4—H4	120.4
C2—C3—C6	119.70 (15)	N5—C1—C2	119.82 (16)
N4—C6—N1	112.45 (15)	N5—C1—H1	120.1
N4—C6—C3	123.79 (15)	C2—C1—H1	120.1
N1—C6—C3	123.66 (15)		

### Hydrogen-bond geometry (Å, °)

**Table:**

*D*—H···*A*	*D*—H	H···*A*	*D*···*A*	*D*—H···*A*
N5—H5A···O3^i^	0.86	2.28	2.964 (2)	136
N5—H5A···N2^ii^	0.86	2.38	3.059 (2)	136
N3—H3A···O4^iii^	0.86	2.21	2.884 (2)	135

Symmetry codes: (i) −*x*, −*y*+1, −*z*+1; (ii) −*x*+1/2, *y*−1/2, −*z*+3/2; (iii) *x*+1/2, −*y*+3/2, *z*+1/2.
